# Host phylogeny matters: Examining sources of variation in infection risk by blood parasites across a tropical montane bird community in India

**DOI:** 10.1186/s13071-020-04404-8

**Published:** 2020-10-28

**Authors:** Pooja Gupta, C. K. Vishnudas, V. V. Robin, Guha Dharmarajan

**Affiliations:** 1 Savannah River Ecology Laboratory, University of Georgia, Aiken, SC USA; 2 Warnell School of Forestry and Natural Resources, University of Georgia, Athens, GA 30602 USA; 3 Indian Institute of Science Education and Research Kolkata, Mohanpur, West Bengal 741246 India; 4 Indian Institute of Science Education and Research Tirupati, Mangalam, Tirupati 517507 India

**Keywords:** Avian haemosporidians, *Plasmodium*, *Haemoproteus*, Ecological traits, Host phylogeny, Infection dynamics, Western Ghats, India

## Abstract

**Background:**

Identifying patterns and drivers of infection risk among host communities is crucial to elucidate disease dynamics and predict infectious disease risk in wildlife populations. Blood parasites of the genera *Plasmodium* and *Haemoproteus* are a diverse group of vector-borne protozoan parasites that affect bird populations globally. Despite their widespread distribution and exceptional diversity, factors underlying haemosporidian infection risk in wild bird communities remain poorly understood. While some studies have examined variation in avian haemosporidian risk, researchers have primarily focused on host ecological traits without considering host phylogenetic relationships. In this study, we employ a phylogenetically informed approach to examine the association between host ecological traits and haemosporidian infection risk in endemic bird communities in the Western Ghats Sky Islands.

**Methods:**

We used parasite sequence data based on partial mitochondrial cytochrome *b* gene, that was amplified from genomic DNA extracted from 1177 birds (28 species) across the Western Ghats to assess infection of birds with haemosporidian parasites. We employed a Bayesian phylogenetic mixed effect modelling approach to test whether haemosporidian infection risk was affected by seven species-specific and four individual-level ecological predictors. We also examined the effect of host phylogenetic relationships on the observed patterns of variation in haemosporidian infection risk by estimating phylogenetic signal.

**Results:**

Our study shows that host ecological traits and host phylogeny differentially influence infection risk by *Plasmodium* (generalist parasite) and *Haemoproteus* (specialist parasite). For *Plasmodium*, we found that sociality, sexual dimorphism and foraging strata were important ecological predictors. For *Haemoproteus*, patterns of infection risk among host species were associated with sociality, species elevation and individual body condition. Interestingly, variance in infection risk explained by host phylogeny was higher for *Haemoproteus* parasites compared to *Plasmodium*.

**Conclusions:**

Our study highlights that while host ecological traits promoting parasite exposure and host susceptibility are important determinants of infection risk, host phylogeny also contributes substantially to predicting patterns of haemosporidian infection risk in multi-host communities. Importantly, infection risk is driven by joint contributions of host ecology and host phylogeny and studying these effects together could increase our ability to better understand the drivers of infection risk and predict future disease threats.

**Graphical abstract:**

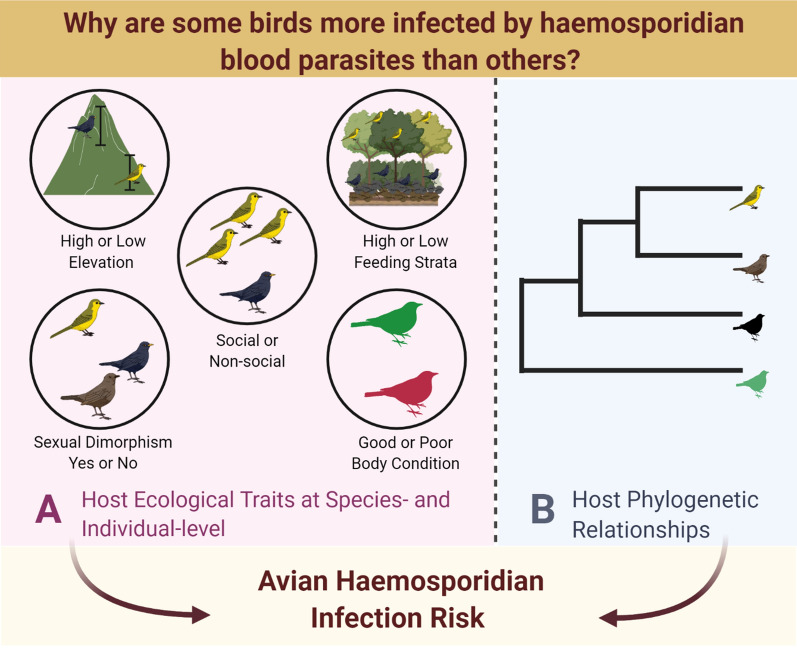

## Background

Identifying factors that determine the variation in infection risk in natural populations is of fundamental importance for understanding the ecology and evolution of host-parasite interactions, predicting infection risk and biological conservation [1, 2]. In multi-host, multi-parasite systems, a myriad of factors operating at the individual- and species-level can affect the probability of parasite exposure and subsequent infection across host species [3–6]. At the species level, variation in infection risk can occur because of differences in host life history, behavior and environment that underpin patterns of parasite exposure [7–10]. At the individual host level, hosts can vary in infection risk owing to differences in exposure to parasites and host susceptibility. In the case of vector-borne diseases, hosts exposure to parasites can increase *via* increase in frequency of encounter with dipteran vectors that can influence disease transmission [11]. For instance, host exposure can be impacted by geographical factors that affect vector abundance (e.g. elevation [12]) or host ecological traits that affect exposure risk such as foraging/nest height [13, 14] or sociality [10, 15, 16]. Additionally, some species-specific traits associated with disease susceptibility (e.g. sexual dimorphism) or individual-level traits associated with fitness (e.g. fluctuating asymmetry [17, 18] and body condition [19, 20]) could be important predictors of infection risk in natural communities.

Avian haemosporidian parasites (Apicomplexa: Haemosporida) of the genera *Plasmodium*, *Haemoproteus* (including *Parahaemoproteus*) are protozoan blood parasites that affect bird populations globally [21]. Avian haemosporidians (commonly referred to as avian malaria parasites) are an exceptionally diverse group of parasites, with over 2500 parasite genetic lineages [22]. These parasites are transmitted by arthropod vectors, with *Plasmodium* being transmitted by culicid mosquitoes, and *Haemoproteus* by ceratopogonid biting midges and hippoboscid louse flies [21, 23]. Avian haemosporidians can impose strong selective pressures on bird hosts as they are known to reduce longevity [24], host fitness [25, 26], individual host condition [27] and have led to severe population declines [28–31]. Previous research has revealed that avian haemosporidian parasites vary widely in their host range, with *Plasmodium* lineages often being generalists infecting a broad range of host species and *Haemoproteus* lineages often being specialists infecting one or few closely related host species [32–34] but this pattern is not universal [35, 36]. *Plasmodium* and *Haemoproteus* parasites are also known to exhibit eco-evolutionary differences, with community structure of *Plasmodium* generally affected by abiotic factors such as spatial proximity and *Haemoproteus*, primarily affected by biotic factors such as host phylogeny and host ecology [32, 37, 38] but see [39, 40]. Given the widespread distribution, diversity and pronounced eco-evolutionary differences between *Plasmodium* and *Haemoproteus*, variation in parasite prevalence for the two parasite genera could be affected by different ecological and evolutionary factors in multi-host communities.

Several studies have attempted to identify ecological factors that can predict haemosporidian infection risk in avian communities, but offer mixed support, in part, owing to the limited exploration of concomitant factors simultaneously across entire host assemblages and across both parasite genera or challenges associated with understanding complex interactions operating across different scales (e.g. within and between species). For instance, specific habitat and temperature requirements of different haemosporidian vectors (e.g. mosquitoes, biting midges) and parasites may limit their distribution on an elevational gradient and across habitat types [12, 41]. While some studies support higher prevalence of *Haemoproteus* at high elevations and high prevalence of *Plasmodium* at lower elevations [42, 43] others present contrasting patterns, with high prevalence of *Haemoproteus* and *Plasmodium* at mid-elevations [44] and no effect of elevation for *Haemoproteus* and *Plasmodium* parasites [14].

Roosting and foraging stratum of host species may increase the probability of hosts encountering vectors thereby promoting parasite transmission. It has been hypothesized that social aggregations attract vectors, and this could lead to higher prevalence of avian haemosporidians in social species [16, 45]. Furthermore, vertical stratification in arthropod vectors could result in variation in avian haemosporidian infection risk due to differences in abundance of vectors in the canopy compared to the ground level [46–48]. Other species ecological traits such as host specialization (e.g. habitat or elevational specialization) could also lead to differential exposure to vectors/parasites. Generalists species, spanning a wider elevational range, may encounter more vectors or a greater diversity of habitats compared to elevational specialists, leading to higher parasite prevalence in generalists than specialists. Furthermore, previous studies suggest host species that exhibit sexually dimorphic traits (e.g. plumage brightness) have likely been exposed to higher levels of parasitism because parasite pressure is a strong driver of sexual selection on these traits [49, 50]. Thus, species that are more susceptible to parasite infections likely exhibit higher levels of sexual dimorphism [49].

At the individual level, previous studies suggest that birds with higher average body size tend to have higher infection probability as larger body size will likely provide more surface area for vector feeding and emit higher quantity of olfactory cues (e.g. CO_2_), thereby attracting more vectors [14, 51]. Host body condition can also affect the likelihood of infection due to differences in individual susceptibility. A negative association between body condition and parasitism is generally expected, either due to reduced immunocompetence in birds with poor body condition and increased susceptibility or the direct effects of parasitism on the fitness of individuals, leading to poor body condition [4, 20]. Furthermore, fluctuating asymmetry (defined as small, random deviations from symmetry of bilateral symmetrical traits [52]) could be an important predictor for avian haemosporidian infection. The positive association between fluctuating asymmetry and parasitism is relatively common in natural populations [17, 18], and this association could exist either because parasitism (as a form of developmental stress) can directly increase levels of fluctuating asymmetry, or because individuals with high fluctuating asymmetry have low immunity.

Although several factors have been proposed to explain variation in parasite prevalence and infection risk among individuals and host species [13, 14, 43, 48, 51, 53], it remains unclear whether the role of host ecological traits are generally predictable or whether they are idiosyncratic across hosts, parasites and environmental conditions and context-dependent. Additionally, evolutionary history of host species can confound the relationship between ecological traits and parasite infection risk as closely related species are more likely to share risk factors compared to non-related host species. Despite this, surprisingly few studies have taken evolutionary history of the hosts into account (e.g. [48, 54]) and thus, the importance of host evolutionary history in predicting infection risk is less well understood.

**Fig. 1:**
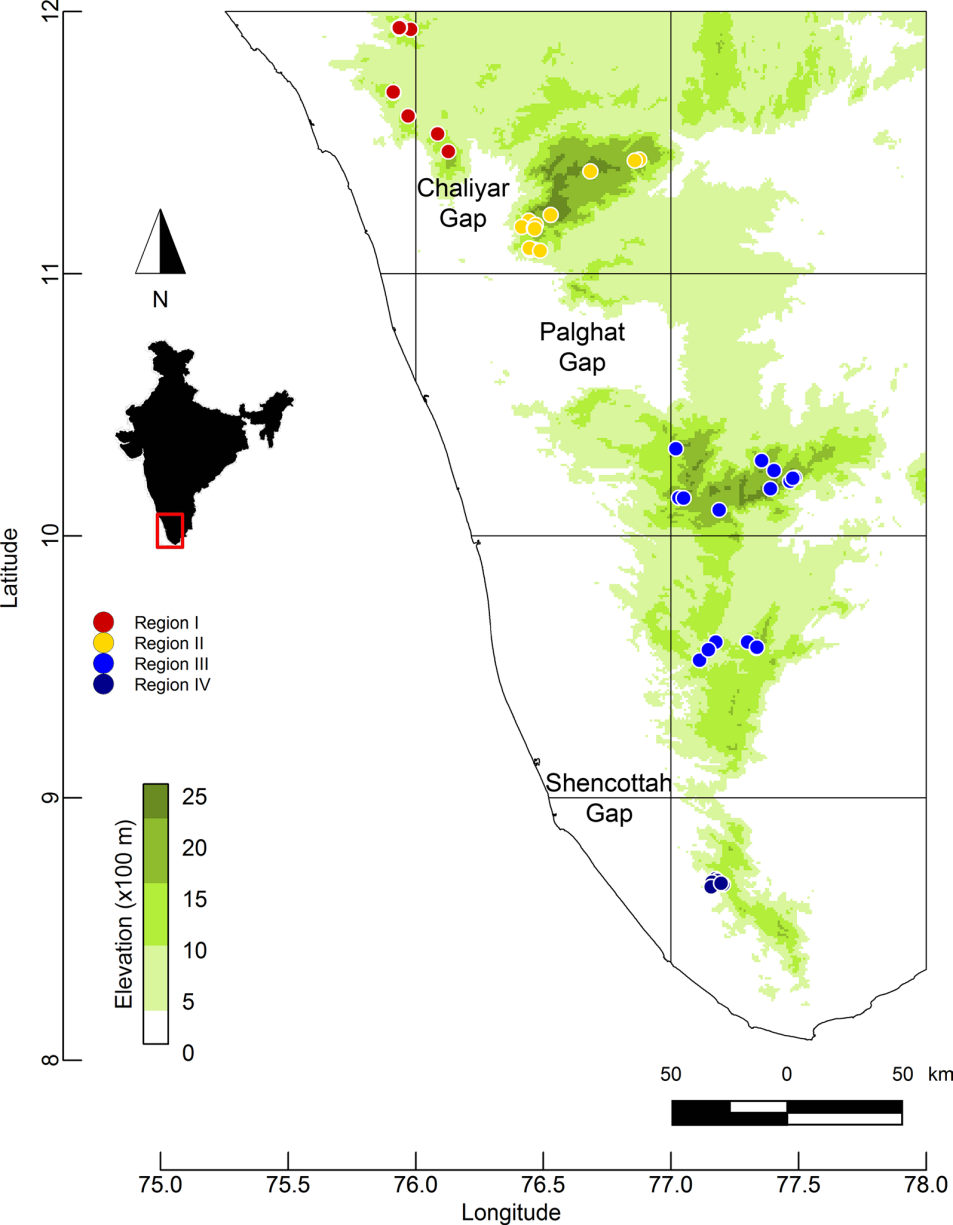
Map of the Western Ghats including locations of sampling sites (filled circles) in four geographical regions: I (Bababudan and Banasura hills), II (Nilgiri hills), III (Anamalai-Palni-Highwavies hills), IV (Ashambu hills), corresponding to the major Sky Island group. Underlying elevation gradient in the Western Ghats is also depicted, with Shola Sky Islands located above 1400 m.a.s.l

The Tropical Sky Island bird community in the Western Ghats mountains, located parallel to the southern coast of India (Fig. 1), offers an excellent model system to elucidate the factors influencing variation in avian haemosporidian infection risk. The Western Ghats are a global biodiversity hotspot [55], and the high endemic bird diversity in the Western Ghats [56] provides opportunities for native parasites to exploit a wide variety of hosts, allowing us to test how host ecology impacts parasite infection risk. Additionally, the landscape is threatened by anthropogenic habitat fragmentation and land-use changes; and the potential negative impact of avian malaria in this biodiversity hotspot makes the identification of factors associated with increased infection risk an important step for conservation [57].

Sky Islands are isolated mountain-top habitats surrounded by dramatically different lowland habitats. The replicated arrangement of geographically discrete, identical habitats provides an ideal natural laboratory to explore ecological dynamics underlying avian haemosporidian infection risk. The Western Ghats mountains are interrupted by three major bio-geographical breaks, the Chaliyar River valley (2–3 km wide), the deepest Palghat Gap (40 km wide) and the Shencottah Gap (10 km wide), resulting in genetic differentiation and speciation across a range of taxa [58–60]. Such patterns of genetic differentiation in hosts could impact the spatial distribution of parasite populations harboring these bird hosts. Within each Western Ghats mountain, Sky Islands hosts unique natural matrix of wet, montane evergreen forests and grasslands, locally known as *Sholas,* above 1400 m (henceforth Shola Sky Islands), while low elevations harbor drier habitats. High habitat heterogeneity and climatic conditions due to its elevational gradient have led to disproportionately high host species diversity in the Shola Sky Islands, comprising of host species having different habitat specialization, life history strategies and elevational distribution. For example, montane specialists are restricted to high elevations and generalists are distributed widely from high to low elevations. While montane specialists have likely been historically protected from avian malaria because low temperatures at high elevations leads to low vector abundance [61] or poor parasite development [12], this scenario might be changing as global warming progresses [41, 62]. Thus, Western Ghats Sky Islands offer a valuable system in which to investigate infection dynamics, especially in the light of possible climate change driven extinctions in the landscape (e.g. Robin et al. [63]).

**Fig. 2:**
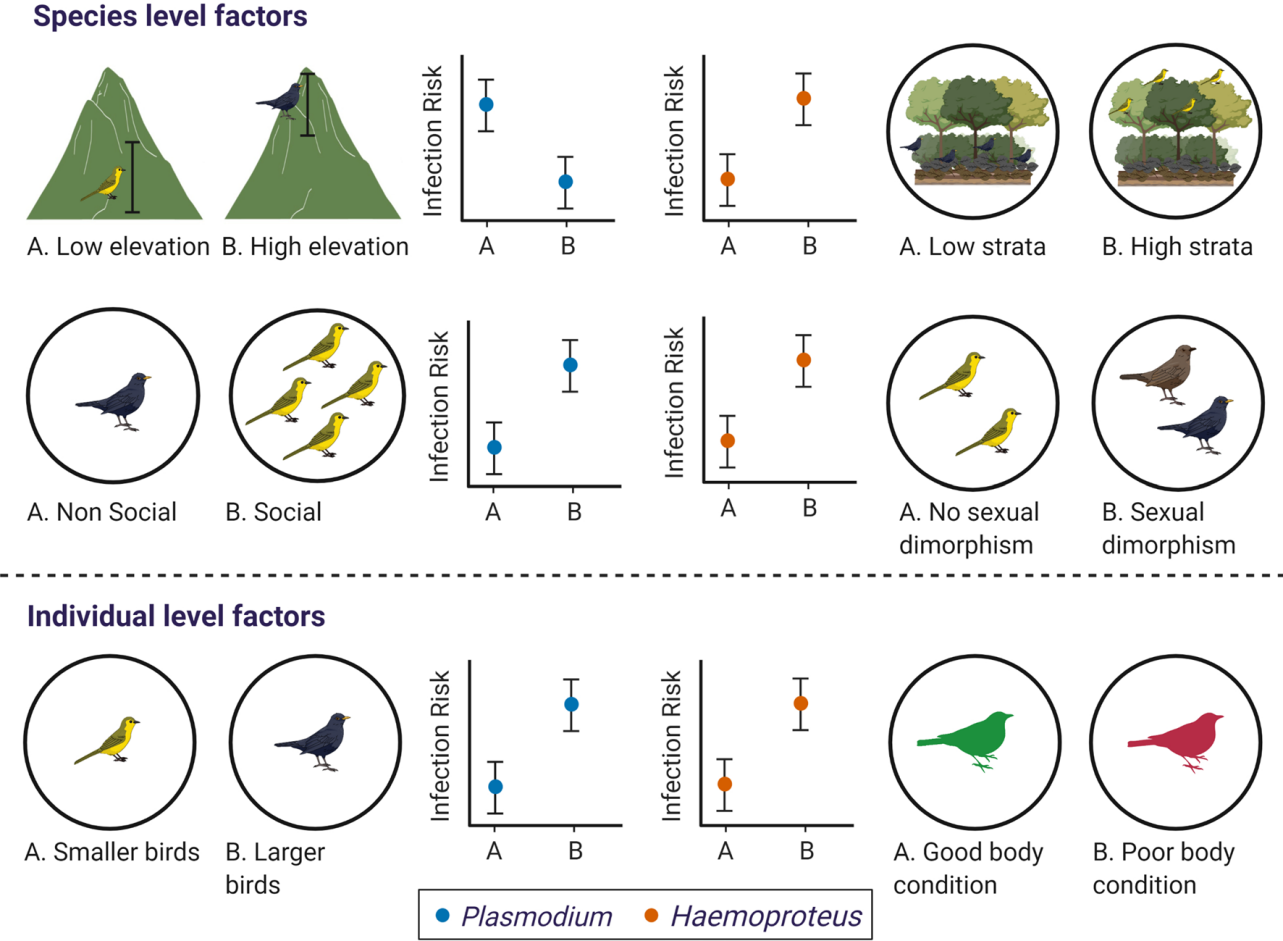
Predictions for the expected effects of different host ecological traits at the species-level and individual-level on infection risk by *Plasmodium* and *Haemoproteus* parasites. Plots show hypothetical relationships between infection risk (*Plasmodium*, blue and *Haemoproteus*, orange) and each level (A and B) of a particular ecological predictor; common plots shown for two ecologcial predictors on each row

In this study, we first examine the species- and individual-level ecological factors that influence variation in avian haemosporidian prevalence and thus avian haemosporidian infection risk in the Western Ghats. Next, we examine if these effects differ across the two parasite genera, *Plasmodium* and *Haemoproteus*. Secondly, we test the effect of host evolutionary history in explaining variation in avian haemosporidian infection risk not explained by host ecological factors. As mentioned earlier, several studies suggest that *Plasmodium* is a generalist parasite and *Haemoproteus* is a relatively specialist parasite [32–34], thus we expect that the effects of ecological factors will vary for *Plasmodium* and *Haemoproteus* in addition to their intrinsic differences in parasite biology and vector specificity. At the species-level, we expect that: (i) species that have a lower minimum elevation will have higher *Plasmodium* prevalence whereas species with a higher minimum elevation will have higher *Haemoproteus* prevalence (Fig. 2); (ii) species foraging at higher forest strata will have lower *Plasmodium* prevalence and higher *Haemoproteus* prevalence compared to species foraging at the ground level (Fig. 2); (iii) social living species and species with sexually dimorphic traits will likely exhibit higher parasite prevalence of both parasites (Fig. 2). Furthermore, at the individual-level, we expect: (iv) infection risk would increase with increase in body size and fluctuating asymmetry (Fig. 2); and (v) birds with better body condition will likely be less infected by both parasites compared to birds with poorer body condition (Fig. 2).

## Methods

### Study area and collection of parasite data

We used bird data and parasite genetic data collected as part of an earlier study, see details in Gupta et al. [32]. Briefly, birds were captured using mist-nets at 52 localities across four major Sky Island groups, separated by three biogeographic barriers, spanning 600 km in the southern Western Ghats mountain range during 2011–2013 (Fig. 1). Blood samples (50–100 μl) were collected from the ulnar vein of the bird with a heparinized micro-hematocrit capillary tube and immediately stored in blood lysis buffer. Avian haemosporidian infection was identified by amplifying parasite’s partial mitochondrial cytochrome *b* gene (478 bp) [64]. Positive infections were sequenced, and paired DNA sequences were aligned in Geneious 9.1.5 [65]. Unique haemosporidian lineages were identified by comparing parasite sequences with publicly available sequences in NCBI and in the MalAvi database [22].

### Ecological trait data

We collected data on host ecological traits based on the current understanding of vectors transmitting avian haemosporidians and included traits that increase hosts’ exposure and/or host susceptibility to parasitism. Data on ecological traits of host species was collected from previous field observations by CKV and VVR and additionally sourced from the Wilman dataset [66]. Our dataset included seven species-specific variables: foraging strata (high/low), roosting behavior (social/non-social), host habitat type (forest/grassland), elevational range (specialist/generalist), genetic connectivity (breaks/no breaks), sexual dimorphism (yes/no) as categorical variables and minimum species elevation as a covariate (details in Additional file 1: Tables S1, S2). Briefly, birds were classified into two categories based on their foraging strata: low (ground foraging) and high (understory to mid-level foraging) based on Somasundaram et al. [67] and Wilman et al. [66], with the former being given precedence as it reported data specific to the Western Ghats. Species roosting behavior was categorized into social and non-social based on field observations by CKV and VVR. Host habitat type was based on species habitat preferences: forest (species preferring evergreen, semi-evergreen, moist-deciduous, dry-deciduous, scrub habitat) or grassland (open country, grassland) based on Ali & Ripley [68] and field observations by CKV and VVR. Species elevational range was classified into two categories: specialists (species restricted to high-elevations, occurring above 1400 m) and generalists (species having a wider distribution ranging from low to high elevations) as assessed in an earlier study [58]. Genetic connectivity was classified as: breaks/no breaks (species with evidence of genetic divergence or no evidence of genetic divergence due to the biogeographical gaps, respectively) based on Robin et al. [58]. Sexual dimorphism was classified into two categories: yes/no (species with differences in plumage between sexes or no plumage differences between sexes, respectively) based on Ali & Ripley [68]. We used bird distribution data to estimate the minimum elevational distribution extent of the bird species (details in Additional file 1: Table S2).

At the level of individual host, the ecological trait data consisted of four variables associated with body size, fluctuating asymmetry (FA) and body condition. The body size variables included tarsus and wing measurements (details in Additional file 1: Table S2). We calculated a measure of fluctuating asymmetry with respect to tarsus (FA_Tarsus_) as per Van Dongen [69]. We also estimated individual body condition, a commonly used proxy of infection-induced fitness cost [20], based on scaled mass index $\hat{M},$ as proposed by Peig & Green [70], which accounts for covariance between body size and body mass components. The condition score was calculated by standardizing body mass at a fixed value of a linear body measurement based on the scaling relationship between mass and length. We used body weight as the mass measurement and wing length measurement as the length variable because average wing length was most strongly correlated with body weight on a log-log scale (Pearson correlation, *r* = 0.80, *P* < 0.001, details in Additional file 1: Table S2). All individual measurement variables were standardized by a z-transform within each species (i.e. a unit increase in the measurement indicates one standard deviation increase over the mean value for the species).

### Statistical analyses

We built Bayesian phylogenetic mixed models (BPMM) to assess the association between infection risk and host ecological and morphometric traits using the R-package *MCMCglmm* [71]. We used BPMM as it allowed us to control for statistical non-independence of trait data due to host phylogenetic relationships [72]. We modeled host infection status as a binary response variable (0 for uninfected, 1 for infected) with a logit link, for *Plasmodium* and *Haemoproteus*, and different species- and individual-level ecological traits as predictor variables. To account for shared ancestry between host species, we fitted a variance-covariance matrix of phylogenetic distances between host species generated from the host phylogeny as a random effect. We used host phylogeny based on cytochrome *b* sequence data (1143 bp) from earlier studies [32, 58]. We included sampling sites as another random effect to account for non-independence among the sampled individuals due to sampling design. We conducted two separate BPMM analyses with the host species ecological traits and individual trait data because we had complete morphometric measurements for only a subset of individuals (*n* = 991 individuals). We excluded all individuals without complete information from the individual level BPMM analysis.

For both datasets, we first tested a fully parameterized model including all predictors and then ran subsequent reduced models by excluding non-significant predictors, one at a time based on *P*-values. We used weak, uninformative prior (normal distribution with mean of zero and very large variance) for the fixed effects, an expanded prior (*χ*^2^ distribution with 1 degree of freedom) for the random effects and fixed residual variance at 1, based on recommendations by de Villemereuil et al. [73] and Hadfield [71]. We ran each model chain for 2 million iterations with burn-in of 100,000 and thinning intervals of 1000 iterations. Additionally, we conducted three independent MCMC runs for our final reduced model that included significant predictors from both species- and individual-level analyses. Analyses for each parasite genus (*Plasmodium* and *Haemoproteus*) were conducted separately.

We visually analyzed the trace plots for all model parameters to assess mixing properties and stationarity of chains. We assessed convergence of the MCMC chains by evaluating correlation between samples (autocorrelation < 0.1) and Gelman-Rubin statistic (potential scale reduction factor, PSRF < 1.1 preferred among chains) using R-package *coda* [74]. We considered model parameters to be significant when the 95% credible intervals (CIs) of posterior estimates excluded zero and *P*-values were < 0.05. We evaluated the performance of our final reduced models using a suite of standard metrics including sensitivity, specificity, and area under the receiver-operator curve (AUC) statistic, as implemented in the R-package *cutpointr*. We also calculated predicted infection probabilities for each species and evaluated the model fits by plotting the observed and predicted infection probabilities.

Furthermore, we calculated the proportion of the total variance explained by host species phylogeny by estimating phylogenetic heritability, equivalent to Pagel’s lambda (*λ*) to measure the degree of phylogenetic signal [75, 76]. We estimated the mean and 95% highest posterior density (HPD) of *λ* for each MCMC chain by dividing the phylogenetic variance-covariance (VCV) matrix by the sum of the phylogenetic, location, and residual VCV matrices [76]. All statistical analyses and graphing were conducted in R ver. 3.6.2 [77].

## Results

### Avian haemosporidian prevalence

Our dataset included 1177 birds across 28 bird species, representing almost the entire Shola Sky Island bird community (Additional file 1: Table S1). We found 24/28 bird species infected (490 birds, 41.6% prevalence) with avian haemosporidians. Among the 47 unique haemosporidian lineages, 10/18 *Plasmodium* and 24/29 *Haemoproteus* lineages were novel and endemic to the Shola Sky Islands [32]. Haemosporidian prevalence varied across host species, with *Turdus merula* exhibiting high *Plasmodium* prevalence (29%, *n* = 86) and *Zosterops palpebrosus* showing high *Haemoproteus* prevalence (77.1%, *n* = 118). The evaluation metrics revealed that final reduced models fit the data well in the case of both *Plasmodium* (sensitivity = 0.788; specificity = 0.842; AUC = 0.874; Additional file 1: Table S4) and *Haemoproteus* (sensitivity = 0.889; specificity = 0.709; AUC = 0.869; Additional file 1: Table S4) parasites. We also observed a strong association between the observed infection probability and predicted infection probability for each species across both genera (*Plasmodium*, *R*^2^ = 0.88, *P* < 0.001; *Haemoproteus*, *R*^2^ = 0.95, *P* < 0.001), suggesting that our sampling was adequate to capture the true prevalence for each species (Additional file 1: Figure S1).

### Species ecology and individual body condition affects avian haemosporidian prevalence

**Fig. 3:**
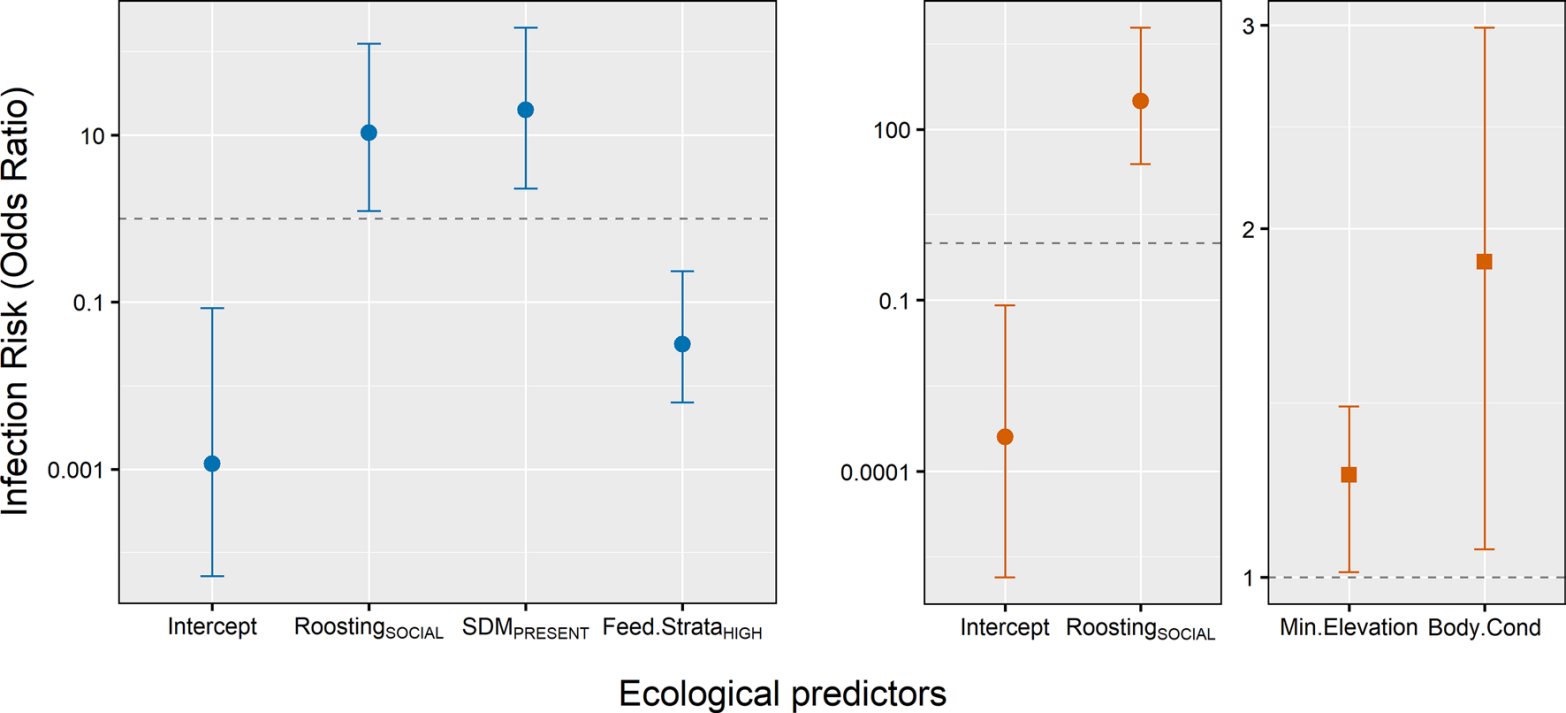
The effect of host ecological predictors on avian haemosporidian infection risk in the Western Ghats Sky Islands. Results of our final reduced Bayesian phylogenetic mixed model with posterior mean estimates and 95% credible intervals (CIs) of all significant predictors on infection risk by *Plasmodium* (**a**) and *Haemoproteus* (**b**). Model parameters were considered significant when the 95% CIs of posterior estimates excluded zero. Categorical variables tested include roosting behavior (non-social *vs* social), sexual dimorphism (absent *vs* present), feeding strata (low *vs* high), with the former as the reference category and two covariates: species minimum elevation and individual body condition (scaled mass index)

Some ecological predictors that we tested were unimportant for infection status responses (i.e. the 95% CI overlapped with 0) and were removed to construct the reduced models (Additional file 1: Table S3). As expected, different ecological predictors were important for variation in infection risk by *Plasmodium* and *Haemoproteus.* At the species level, sociality and sexual dimorphism were positively associated with *Plasmodium* prevalence (*β* = 2.56, CI: 0.30–5.06; OR: 10.6) and (*β* = 3.11, CI: 0.84–5.19; OR: 20.1), respectively (Fig. 3a). Additionally, species foraging at high strata had lower *Plasmodium* prevalence (*β* = -3.29, CI: -5.07– -1.45; OR: 0.03) compared to low strata foragers. For *Haemoproteus*, sociality and species elevation were significant predictors of *Haemoproteus* parasite prevalence in the Shola Sky Island bird community (Fig. 3b, Additional file 1: Table S3). Social roosting species had higher *Haemoproteus* prevalence (*β* = 5.91, CI: 3.26–8.67; OR: 315.5) compared to non-social species. The exceptionally high odds ratio for social *vs* non-social species is interesting and reflects the large observed difference in parasite prevalence between these two groups (0.53% and 0.13%, respectively). Minimum elevation of host species had a significant positive association with *Haemoproteus* prevalence (*β* = 0.17, CI: 0.01–0.34; OR: 1.22).

Among the individual level predictors, we did not find significant relationship between the various morphometric traits (tarsus and wing lengths), fluctuating asymmetry, body condition and variation in haemosporidian prevalence for *Plasmodium* parasites. But our final model for *Haemoproteus* revealed individual body condition as a significant predictor for *Haemoproteus* prevalence (Fig. 3b, Additional file 1: Table S3). Surprisingly, *Haemoproteus* prevalence increased significantly with birds having better body condition (*β* = 0.59, CI: 0.07–1.12; OR: 1.87). All other predictors revealed no significant relationship with *Haemoproteus* parasite prevalence.

### Phylogenetic signal

**Fig. 4:**
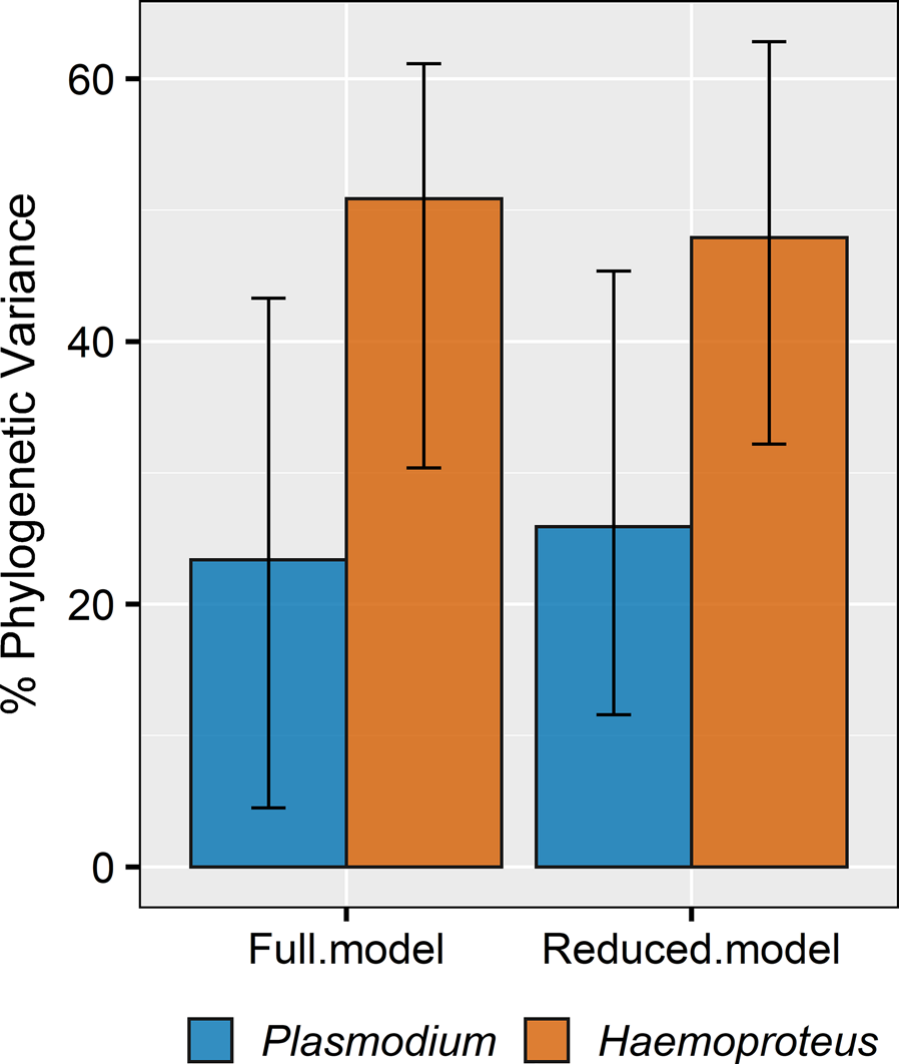
Proportion of total variance attributed to host species phylogeny representing phylogenetic signal or lambda (k). Reported are the percent posterior means and 95% credible intervals across full and reduced Bayesian phylogenetic mixed models estimated in MCMCglmm, shown for *Plasmodium* (blue) and *Haemoproteus* (orange)

We recovered phylogenetic signal in both our full and reduced models, in the case of both *Plasmodium* and *Haemoproteus*; however, phylogenetic signal was lower for *Plasmodium* compared to *Haemoproteus*. After taking into account the variation explained by host ecological traits, location effects and residual variance, host species phylogeny explained 27% (*β* = 4.78, CI: 1.29–8.95) of the total variation observed in *Plasmodium* prevalence and 48% (*β* = 10.97, CI: 5.14–18.42) of the total variation in *Haemoproteus* prevalence across host species (Fig. 4, Additional file 1: Table S5).

## Discussion

In this study, we show that multiple host ecological factors are important determinants of haemosporidian infection risk across avian hosts in the Western Ghats Sky Island bird community. However, these effects varied among *Plasmodium* and *Haemoproteus* parasites, likely due to their eco-evolutionary differences and vector preferences. Previous studies have also reported mixed support for the ability of host ecological factors to predict avian haemosporidian prevalence [14, 43, 48, 51]. This suggests that these patterns are far from universal and underlying host community structure influencing parasite exposure and/or host evolutionary history influencing host susceptibility likely plays a key role in assessing avian haemosporidian infection risk.

We hypothesized that prevalence of *Haemoproteus* parasites will increase with species’ elevation as cooler temperatures at higher elevations may support the survival and development of both *Haemoproteus* parasites and their associated vectors (i.e. biting midges [78]). We lend support to this hypothesis as our results show that species elevation was significantly associated with *Haemoproteus* prevalence, with species at higher minimum elevation having higher *Haemoproteus* prevalence compared to species at lower minimum elevation. This agrees with previous studies that had found support for higher *Haemoproteus* prevalence at higher elevations with low temperatures [43, 79, 80]. In the light of global climate change, our findings indicate that *Haemoproteus* parasites, which are currently more prevalent at higher elevations, might undergo range collapse due to unavailability of suitable environment (niche) for its survival and development [81]. In addition to the environmental constraints on *Haemoproteus* parasites, the observed patterns could also be confounded by specific host species present at high elevation as *Haemoproteus* parasites tend to be host specialists in the Western Ghats [32].

Our findings indicate that host species ecological traits that promote exposure risk likely explain the increased prevalence of avian haemosporidian parasites. Several studies have found evidence for higher avian haemosporidian prevalence in social birds [45], but see Arriero & Moller [15]. Among the various host ecological traits tested in our study, we found sociality as a consistent and an important explanatory variable, positively associated with prevalence of both *Plasmodium* and *Haemoproteus*. Sociality may increase the probability of hosts encountering vectors thereby promoting parasite transmission [6]. It has been hypothesized that higher aggregation of vectors may occur around social species as host-seeking behavior of malaria vectors relies on the odor cues (CO_2_) and chemical attractants released by the host species [82]. This may explain higher prevalence of avian haemosporidians among social species in the Western Ghats Sky Island bird communities.

While there was no significant association between *Haemoproteus* prevalence and foraging strata of host species, species foraging at high strata (canopy level) exhibited lower *Plasmodium* prevalence compared to species at the ground level. Vertical stratification in arthropod vectors that influence hosts’ exposure risk could drive this variation in *Plasmodium* prevalence due to differences in vector abundance. Vectors for *Plasmodium* (*Culex* spp. and *Aedes* spp.) are known to preferentially feed at the ground-level [46, 47, 83], thus reducing their abundance at the canopy level. However, our findings contrast with other studies that showed higher *Plasmodium* prevalence for middle- to high-level foragers [84] and low for ground foragers [85]. Although we could not yet directly assess the role of vectors in transmitting avian haemosporidian parasites in the Western Ghats, we propose integrating information on the distribution and abundance of mosquitoes and biting midges in future research will be invaluable and help resolve these conflicting patterns.

Inter-specific variation in avian haemosporidian prevalence may also result from differences in host susceptibility to infection. Host susceptibility can vary among hosts due to host traits or differences in host-parasite coevolutionary histories [86]. As expected, we found levels of sexual dimorphism were positively associated with *Plasmodium* infection risk, as has been reported in previous studies [85, 87]. This pattern of sexual dimorphism affecting haemosporidian infection lends support to Hamilton and Zukʼs hypothesis [50], whereby sexual selection favors costly male phenotypic traits (e.g. plumage brightness) as indicators of parasite resistance. Thus, higher levels of sexual dimorphism among species tends to be associated with higher parasite infection [49].

With individual body condition, we expected to find low probability of infection in birds with better condition because generally, parasitic infections negatively affect host body condition [20, 27, 88]. Hosts in poor body condition are likely more susceptible to infection due to reduced immunocompetence [4, 89, 90]. Contrary to our expectations, we found no significant association between host body condition and *Plasmodium* infection and a positive effect of body condition on the probability of *Haemoproteus* infection. Birds with better body condition had higher *Haemoproteus* infection compared to birds in poorer body condition. Although parasites are generally thought to be detrimental to their hosts, parasites may not always be harmful to their hosts and hosts in good body condition can often tolerate higher parasite loads, leading to a positive relationship between body condition and infection status [20, 91]. Our findings suggest that birds were likely tolerant to *Haemoproteus* infection and did not suffer high costs to infection and or at least to the extent that it is not reflected in their body condition. However, parasitemia data and other fitness measures (e.g. reproductive success) are needed to confirm our findings of fitness costs and the underlying host defense mechanisms in response to avian haemosporidian infection in the Western Ghats. Understanding the relative investment in resistance *vs* tolerance is critical, as it can affect disease dynamics at both individual- and species-level [92]. For example, highly tolerant individuals could be more efficient at transmitting disease in a population (i.e. super-spreaders [93]). Additionally, host species that are tolerant to parasite infection may serve as reservoirs of infection and represent an indirect threat to more vulnerable host species, as has been shown in other host-parasite systems (e.g. [94–96]), an issue critical for conservation of threatened host species.

We found higher phylogenetic signal in *Haemoproteus* compared to *Plasmodium*, indicating phylogenetic conservatism of host susceptibility to infection. Host phylogenetic relationships could be important in shaping patterns of parasite prevalence and disease transmission because closely related hosts are similar in their behavioral, physiological and immunological characteristics [97]. Consequently, several studies have shown that closely related hosts share more similar parasite communities and host phylogenetic distance is a key predictor of cross-species transmission [98–100], thus, increasing the likelihood for emergence of infectious diseases. Our results on infection dynamics support findings from a previous genetic study which showed that *Haemoproteus* have high phylogenetic host specificity and closely related host species tend to share similar *Haemoproteus* lineages compared to *Plasmodium*, a relatively generalist parasite [32]. Although the magnitude of effect differs between the two parasite genera, we demonstrate that host ecological traits and host evolutionary history are both important factors in explaining interspecific variation in avian haemosporidian infection risk. It is possible that constraints on the distribution of these parasites are likely more related to their avian hosts (not vectors) within the Western Ghats Sky Island bird community. However, a better understanding of the relative importance of ecology of bird hosts and vectors of avian haemosporidians in the Western Ghats will be an important next step to better understand and predict patterns of infection risk for these vector-borne parasites.

## Conclusions

Taken together, we found strong support for the role of host ecological traits and host phylogenetic relationships in influencing variation in avian haemosporidian risk in the Western Ghats Sky Island bird community. As hypothesized, the relative importance of these effects varied among the two avian haemosporidian genera, *Plasmodium* and *Haemoproteus*. Our analyses of various ecological factors suggest that variation in avian haemosporidian infection risk is likely driven by two underlying mechanisms. First, ecological factors (e.g. sociality, foraging strata) that may lead to differential exposure risk could impact avian haemosporidian prevalence. Secondly, ecological factors associated with disease susceptibility or tolerance (e.g. sexual dimorphism, body condition) to infection are important predictors of avian haemosporidian prevalence. To better understand the effect of host ecological factors, research needs to account for host phylogenetic relationships in driving susceptibility to infection and subsequent disease transmission. In this study, we demonstrate the importance of host phylogenetic relationships in influencing variation in infection risk to avian haemosporidians, which is consistent with previous work by Barrow et al. [54]. Higher magnitude of phylogenetic signal in the case of *Haemoproteus* compared to *Plasmodium* parasites tends to be coherent with their host specificity patterns in our community [32]. We conclude that patterns of avian haemosporidian prevalence and infection risk were shaped by joint contributions of both host ecology and host evolutionary history. Understanding host-parasite interactions in a broader eco-evolutionary context, including host phylogenetic relatedness is critical to gain a better understanding of drivers of interspecific variation in infection risk. Ultimately, such efforts could help illuminate the idiosyncratic association between host ecological traits and infection risk, help identify key reservoir hosts and enable improved predictions of infection risk in multi-host communities. Although we focused on avian haemosporidian parasites, this study helps to better understand vector-borne disease dynamics, and particularly improves our understanding of how disease transmission is linked to host behavior (e.g. sociality), physiology (e.g. body condition) and other parameters associated with disease susceptibility (e.g. host evolutionary history). Our results contribute to a growing body of evidence from other vector-borne diseases (e.g. West Nile virus infection and/or Lyme disease) that highlight the importance of host species heterogeneity in disease transmission dynamics [3, 101]. Finally, our work presents an important step towards identifying and understanding variation in infection risk at the individual- and species-level in an important biodiversity hotspot. Elucidating the ecological and evolutionary drivers that contribute to host heterogeneity in infection risk and potential spillover risk to naïve hosts will be important from wildlife health and conservation perspective as the number and severity of emerging infections increase globally.

## Supplementary information

**Additional file 1: Table S1.** Details on the ecological traits for each avian host species sampled from the Shola Sky Island bird community in the Western Ghats. **Table S2.** Detailed methods for calculation of specific ecological and morphological variables used in the Bayesian phylogenetic mixed model (MCMCglmm analysis). **Table S3.** Summary of results for Bayesian phylogenetic mixed models (MCMCglmm analysis) with parasite infection status as the response variable and various host ecological and morphological traits as the predictor variables. **Table S4.** Summary of model evaluation metrics calculated by comparing the observed and predicted data. **Table S5.** Phylogenetic signal or lambda (k) estimates from MCMCglmm for full and reduced models for *Plasmodium* and *Haemoproteus*, calculated as the proportion of total variance attributed to phylogenetic variance. **Figure S1.** Fit between the observed and predicted probabilities of haemosporidian infection for each bird species.

## Data Availability

All data generated or analyzed during this study are included in this published article and its additional files.
