# Correction to: Host phylogeny matters: Examining sources of variation in infection risk by blood parasites across a tropical montane bird community in India

**DOI:** 10.1186/s13071-020-04508-1

**Published:** 2021-02-08

**Authors:** Pooja Gupta, C. K. Vishnudas, V. V. Robin, Guha Dharmarajan

**Affiliations:** 1 Savannah River Ecology Laboratory, University of Georgia, Aiken, SC USA; 2 Warnell School of Forestry and Natural Resources, University of Georgia, Athens, GA 30602 USA; 3 Indian Institute of Science Education and Research Kolkata, Mohanpur, West Bengal 741246 India; 4 Indian Institute of Science Education and Research Tirupati, Mangalam, Tirupati, 517507 India

## Correction to: Parasites Vectors 13:536 (2020) 10.1186/s13071-020-04404-8

Following publication of the original article [1], the authors flagged that the Conclusion of the article Abstract had been erroneously omitted from the PDF version of the article.

The Abstract of the PDF has since been corrected in the published article.

The publisher apologizes for any inconvenience caused.
