# Supplementary material for: Host phylogeny matters: Examining sources of variation in infection risk by blood parasites across a tropical montane bird community in India
Source: Parasit Vectors. 2020 Oct 28;13:536. doi: 10.1186/s13071-020-04404-8 (PMC7594458; doi:10.1186/s13071-020-04404-8)
Supplement: Supplementary file 1 — Additional file 1: Table S1. Details on the ecological traits for each avian host species sampled from the Shola Sky Island bird community in the Western Ghats. Table S2. Detailed methods for calculation of specific ecological and morphological variables used in the Bayesian phylogenetic mixed model (MCMCglmm analysis). Table S3. Summary of results for Bayesian phylogenetic mixed models (MCMCglmm analysis) with parasite infection status as the response variable and various host ecological and morphological traits as the predictor variables. Table S4. Summary of model evaluation metrics calculated by comparing the observed and predicted data. Table S5. Phylogenetic signal or lambda (k) estimates from MCMCglmm for full and reduced models for Plasmodium and Haemoproteus, calculated as the proportion of total variance attributed to phylogenetic variance. Figure S1. Fit between the observed and predicted probabilities of haemosporidian infection for each bird species. [file 13071_2020_4404_MOESM1_ESM.docx]

**TABLES**

**Table S1.** Ecological traits for each avian host species sampled from the Shola Sky Island bird community in the Western Ghats during 2011-2013. Reported are the common and scientific names of each species sampled, sample size (N_TOT_), number of individuals infected with haemosporidian parasites (N_INF_), proportion of infected individuals for *Plasmodium* (P) and *Haemoproteus* (H). The ecological factors reported include: roosting behavior (Social or Non-social), feeding strata (High or Low), habitat type (Forest or Grassland), levels of sexual dimorphism (Yes or No), phylogeographic connectivity of the species, minimum elevation at which the species has been reported.

| **Scientific Name** | **Common Name** | **N_TOT_** | **N_INF_** | **Prevalence %** | | **Roosting** | **Feeding strata** | **Habitat** | **SDM** | **Connectivity** | **Min. Elevation (m)** |
| --- | --- | --- | --- | --- | --- | --- | --- | --- | --- | --- | --- |
|  |  |  |  | **P** | **H** |  |  |  |  |  |  |
| *Alcippe poioicephala* | Brown cheeked fulvetta | 57 | 25 | 5.26 | 33.33 | Social | High | Forest | No | Breaks | 60 |
| *Anthus nilghiriensis* | Nilgiri pipit | 16 | 0 | 0 | 0 | Non-social | Low | Grassland | No | Breaks | 1900 |
| *Anthus rufulus* | Paddyfield pipit | 5 | 1 | 20 | 0 | Non-social | Low | Grassland | No | No Breaks | 0 |
| *Columba elphinstonii* | Nilgiri wood pigeon | 6 | 2 | 0 | 33.33 | Social | High | Forest | No | No Breaks | 600 |
| *Culicicapa ceylonensis* | Grey- headed flycatcher | 29 | 0 | 0 | 0 | Non-social | High | Forest | No | No Breaks | 700 |
| *Cyornis pallipes* | White bellied blue flycatcher | 17 | 5 | 0 | 29.41 | Non-social | High | Forest | Yes | No Breaks | 60 |
| *Eumyias albicaudatus* | Nilgiri flycatcher | 43 | 3 | 4.65 | 0 | Non-social | High | Forest | Yes | Breaks | 1000 |
| *Ficedula nigrorufa* | Black & orange flycatcher | 106 | 11 | 1.89 | 7.55 | Non-social | High | Forest | Yes | No Breaks | 1300 |
| *Garrulax delesserti* | Wayanad laughingthrush | 38 | 30 | 13.16 | 44.74 | Social | Low | Forest | No | No Breaks | 300 |
| *Hypsipetes leucocephalus* | Black bulbul | 58 | 45 | 12.07 | 51.72 | Social | High | Forest | No | No Breaks | 900 |
| *Iole indica* | Yellow browed bulbul | 25 | 12 | 16 | 12 | Social | High | Forest | No | Breaks | 60 |
| *Montecincla cachinnans* | Nilgiri chilappan | 32 | 19 | 3.13 | 46.88 | Social | High | Forest | No | No Breaks | 1400 |
| *Montecincla fairbanki* | Palani chilappan | 102 | 71 | 2.94 | 52.94 | Social | High | Forest | No | No Breaks | 1400 |
| *Montecincla jerdoni* | Banasura chilappan | 21 | 11 | 4.76 | 47.62 | Social | High | Forest | No | No Breaks | 1400 |
| *Montecincla meridionalis* | Ashambu chilappan | 36 | 19 | 2.78 | 44.44 | Social | High | Forest | No | No Breaks | 1400 |
| *Pellorneum ruficeps* | Puff- throated babbler | 27 | 1 | 3.7 | 0 | Non-social | Low | Forest | No | No Breaks | 60 |
| *Pomatorhinus horsfieldii* | Scimitar babbler | 37 | 8 | 2.7 | 13.51 | Non-social | High | Forest | No | Breaks | 300 |
| *Pycnonotus jocosus* | Red-whiskered bulbul | 26 | 1 | 0 | 3.85 | Non-social | High | Forest | No | No Breaks | 0 |
| *Rhopocichla atriceps* | Dark- fronted babbler | 34 | 0 | 0 | 0 | Social | High | Forest | No | Breaks | 60 |
| *Saxicola caprata* | Pied bushchat | 17 | 2 | 5.88 | 5.88 | Non-social | Low | Grassland | Yes | No Breaks | 0 |
| *Schoenicola platyurus* | Broad- tailed grassbird | 7 | 0 | 0 | 0 | Non-social | Low | Grassland | No | No Breaks | 1200 |
| *Sholicola albiventris* | White bellied Sholakili | 100 | 34 | 1 | 32 | Non-social | High | Forest | No | No Breaks | 1400 |
| *Sholicola ashambuensis* | Ashambu Sholakili | 21 | 2 | 0 | 9.52 | Non-social | High | Forest | No | Breaks | 1400 |
| *Sholicola major* | Rufous bellied Sholakili | 86 | 19 | 1.16 | 20.93 | Non-social | High | Forest | No | No Breaks | 1400 |
| *Turdus merula* | Blackbird | 86 | 56 | 29.07 | 6.98 | Non-social | Low | Forest | Yes | Breaks | 1400 |
| *Zoothera citrina* | Orange-headed ground thrush | 10 | 5 | 30 | 0 | Non-social | Low | Forest | Yes | No Breaks | 0 |
| *Zoothera dauma* | Nilgiri thrush | 17 | 11 | 0 | 17.65 | Non-social | Low | Forest | No | No Breaks | 1300 |
| *Zosterops palpebrosus* | Oriental white eye | 118 | 97 | 0.85 | 77.12 | Social | High | Forest | No | Breaks | 700 |

**Table S2.** Detailed methods for the calculation of some specific ecological and morphological variables used in the Bayesian phylogenetic mixed effect model (MCMCglmm analysis).

| **Variable** | **Methodological details** |
| --- | --- |
| ***Species Level Analysis*** | |
| Minimum  Elevation | We used bird distribution data to estimate the Minimum elevational distribution extent of the bird species. The distribution data were collected by collating primary and secondary data, since Indian bird sighting records are not in any database: (i) Primary data: We included sighting records of study species from over a decade’s research in this area including a structured two-year survey by CKV [1], another two-year survey by VVR [2,3] and many field trips by VVR/CKV; (ii) Secondary data: We obtained secondary data from museum records from ORNIS database and also queried checklists and sighting records of three popular birdwatchers e-groups in south India (Bangalore bngbirds -2527 members, Kerala Birder - 918 members and Tamil Birds - 609 members). In cases where exact GPS locations were not indicated in the emails, the locations were assigned on Google Earth in what appeared to be the most appropriate nearby habitat (forest or grassland). These were subsequently extracted to a GIS platform (QGIS Lisboa ver 1.8). Museum collection locations were plotted on Google Earth only when they were unambiguous. We also used the results from a previous search (66) of three search engines (1945 to 2012) - Science Citation Index’s Web of Science (Thomson Reuters, New York, USA), Biological Abstracts (Thomson Reuters, New York, USA) and Google Scholar to collect literature on the study species. We also added information from reports, books, theses and other grey literature using a snowball method (e.g. Nandini & Mudappa [4]). |
| ***Individual Level Analysis*** | |
| Std. Tarsus | Tarsus measurements were taken with Mitutoyo ABS Digimatic Caliper (Mitutoyo Corp Japan) with accuracy of 0.02 mm. Three measurements were taken each from left and right leg to account for measurement error [83]. Std. Tarsus was calculated as the average of all tarsus measurements for each bird standardized by a z-transform within each species (i.e. by subtracting the species mean tarsus measurement from the average measurement of the bird and dividing by the species standard deviation of tarsus measurements). Thus, a unit increase in Std. Tarsus indicates a one SD increase in tarsus length over mean tarsus length for the species. |
| Std. Wing | Wing measurements were taken with a wing rule (wing15econ Avinet Inc., New York, USA) that had a flush stop and calibration from both directions. Three measurements were taken each from left and right wing to account for measurement error [5]. Std. Wing was calculated as the average of all wing measurements for each bird standardized by a z-transform within each species (as described above). |
| Std. FA_Tarsus_ | To estimate the magnitude of fluctuating asymmetry in tarsal measurements, we used the mixed-effects regression model approach analyzed by restricted maximum-likelihood (REML) as proposed by van Dongen et al. [44]. Briefly, we first generate a variable Side (-1 and 1 for left and right tarsusl measurements, respectively). The fixed effects part of the model included Species, Trapping Site and Side, and thus measures the average (i.e. intercept) values of the measurement for each species and site, and estimates the level of directional asymmetry (fixed intercept of the Side variable). The random effects structure included a random intercept for each individual (measuring deviation of the individual from the fixed intercept; see above) and random slope for Side within Individual. The random Side slope measures the magnitude of FA (FA_Tarsus_ ) within each individual (after controlling for other factors; i.e. individual-, species- and site-specific differences in size and magnitude of directional asymmetry). FA estimates were standardized by z-transform within each species (as described above). |
| Std. Body Condition Index | To estimate body condition (CI), we used the scaled mass index (SMI) as proposed by Peig and Green [45]. This index standardizes body mass to a specific fixed value of a linear body measurement based on the scaling relationship between mass and length using the equation: $\hat{M}=M_{i}\left( \frac{L_{0}}{L_{i}} \right)^{b_{SMA}}$where $\hat{M}$ is the scaled body mass, $M_{i}$and $L_{i}$ are the body weight and linear body measurement of individual i, respectively, *b_SMA_* is the scaling exponent estimated by the standardized major axis (SMA) regression of M on L (log-log scale); L_0_ is an arbitrary value of L (e.g., the arithmetic mean value for the study species/population). To estimate *b_SMA_,* we conducted a species-wise linear regression of log-body weight on log-wing length using a type 2 (standaradized major axis regression; SMA) regression. We chose wing measurement as a linear body measurement to scale body weight because average wing length (three each from left and right wing) was most strongly correlated with body weight on a log-log scale (Pearson correlation, r = 0.80, p <0.001); other variable tested was average tarsal length (three each from left and right tarsus). The regression slope values are given in Table S6 below, which were used as a measure of *b_SMA_*. We used average wing measurement for each species as L_0_. Finally, we calculated SMI ($\hat{M})$ as individual body weight × (average wing measurement for each species/average individual wing length) ^ *b_SMA_*. Body CI measurements were standardized by z-transform within each species (as described above). |

**Table S3.** Summary of results for Bayesian phylogenetic mixed models (MCMCglmm analysis) with parasite infection status as the response variable and various host ecological and morphological traits as the predictor variables. Models were run separately for *Plasmodium* and *Haemoproteus*. For each model we report the posterior mean, the 95% credible interval and the p-values. Significant (< 0.05) p-values are in bold.

| ***Plasmodium*** | | | | | | |
| --- | --- | --- | --- | --- | --- | --- |
| ***Species-level*** | **Full model with all predictor variables** | | | **Reduced model with significant effects** | | |
|  | Posterior Mode**^§^** | Lower-95% CI | Upper-95% CI | Posterior Mode**^§^** | Lower-95% CI | Upper-95% CI |
| **Fixed effects** | | | | | | |
| (Intercept) | **-6.129** | -10.171 | -1.327 | **-6.184** | -9.744 | -2.466 |
| ECO_feedStrata02.high | **-3.768** | -6.005 | -1.348 | **-3.292** | -5.074 | -1.454 |
| ECO_socialRoost02.social | **2.158** | -0.081 | 4.380 | **2.550** | 0.306 | 5.063 |
| ECO_sdm02.yes | **2.727** | 0.437 | 4.792 | **3.106** | 0.837 | 5.196 |
| ECO_habitat02.grassland | -2.570 | -6.657 | 0.994 | -- | -- | -- |
| ECO_connect02.breaks | 1.096 | -0.293 | 2.629 | -- | -- | -- |
| ECO_SppWt.div10 | 0.066 | -0.163 | 0.274 | -- | -- | -- |
| ECO_minElev.div100 | -0.005 | -0.135 | 0.127 | -- | -- | -- |
| **Random effects** |  |  |  |  |  |  |
| Species | 3.935 | 0.421 | 8.573 | 4.784 | 1.296 | 8.953 |
| Location | 0.709 | 0.000 | 1.944 | 0.698 | 0.000 | 1.838 |
| ***Individual-level*** | | | | | | |
| (Intercept) | **-6.244** | -11.130 | -1.227 | -- | -- | -- |
| IND_Std.Wing | -0.086 | -0.733 | 0.454 | -- | -- | -- |
| IND_Std.T | -0.202 | -0.832 | 0.444 | -- | -- | -- |
| IND_Std.FA.Tarsus | -0.099 | -0.689 | 0.466 | -- | -- | -- |
| IND_Std.BodyCI | -0.071 | -0.641 | 0.521 | -- | -- | -- |
| **Random effects** |  |  |  |  |  |  |
| Species | 10.573 | 5.171 | 17.643 | -- | -- | -- |
| Location | 1.086 | 0.000 | 2.808 | -- | -- | -- |
| ***Haemoproteus*** | | | | | | |
| ***Species-level*** | **Full model with all predictor variables** | | | **Reduced model with significant effects** | | |
|  | Posterior Mode**^§^** | Lower-95% CI | Upper-95% CI | Posterior Mode**^§^** | Lower-95% CI | Upper-95% CI |
| **Fixed effects** |  |  |  |  |  |  |
| (Intercept) | **-8.972** | -15.857 | -1.297 | **-8.148** | -13.326 | -2.425 |
| ECO_feedStrata02.high | 0.579 | -2.306 | 3.655 | -- | -- | -- |
| ECO_socialRoost02.social | **5.386** | 2.611 | 8.175 | **5.906** | 3.264 | 8.670 |
| ECO_sdm02.yes | -0.372 | -3.214 | 2.561 | -- | -- | -- |
| ECO_habitat02.grassland | -2.187 | -7.815 | 3.134 | -- | -- | -- |
| ECO_connect02.breaks | -0.930 | -2.856 | 1.216 | -- | -- | -- |
| ECO_SppWt.div10 | 0.096 | -0.156 | 0.366 | -- | -- | -- |
| ECO_minElev.div100 | **0.132** | -0.040 | 0.298 | **0.173** | 0.006 | 0.343 |
| **Random effects** |  |  |  |  |  |  |
| Species | 11.517 | 5.012 | 18.983 | 10.975 | 5.141 | 18.425 |
| Location | 1.633 | 0.455 | 3.097 | 0.898 | 0.000 | 2.225 |
| ***Individual-level*** | | | | | | |
| (Intercept) | -5.199 | -11.691 | 0.793 | -8.148 | -13.326 | -2.425 |
| IND_Std.Wing | 0.227 | -0.204 | 0.628 | x | x | x |
| IND_Std.T | -0.382 | -0.817 | 0.004 | x | x | x |
| IND_Std.FA.Tarsus | 0.196 | -0.168 | 0.547 | x | x | x |
| IND_Std.BodyCI2 | **0.715** | 0.256 | 1.127 | **0.599** | 0.070 | 1.119 |
| **Random effects** |  |  |  |  |  |  |
| Species | 17.641 | 9.686 | 26.110 | 10.975 | 5.141 | 18.425 |
| Location | 0.445 | 0.000 | 1.374 | 0.898 | 0.000 | 2.225 |

**§ Bold values indicate bayesian pMCMC Significance <0.05**

**Table S4.** Summary of model evaluation metrics calculated by comparing the observed and predicted data. For each parasite genera, reported are the acc, sensitivity, specificity, and area under the receiver-operator curve (AUC) values.

| **S. No.** | **Parasite spp.** | **acc** | **Sensitivity** | **Specificity** | **AUC** |
| --- | --- | --- | --- | --- | --- |
| 1 | *Plasmodium* | 0.837 | 0.788 | 0.842 | 0.874 |
| 2 | *Haemoproteus* | 0.764 | 0.889 | 0.709 | 0.869 |

**Table S5.** Phylogenetic signal or lambda (k) estimates from MCMCglmm for full and reduced models for *Plasmodium* and *Haemoproteus*, calculated as the proportion of total variance attributed to phylogenetic variance. Reported are the posterior means and 95% credible intervals.

| **Parasite Species** | **Model Type** | **Lambda** (k) | **Lower-95% CI** | **Upper-95% CI** |
| --- | --- | --- | --- | --- |
| *Plasmodium* | Full.model | 0.234 | 0.045 | 0.433 |
| *Plasmodium* | Reduced.model | 0.276 | 0.115 | 0.449 |
| *Haemoproteus* | Full.model | 0.509 | 0.304 | 0.612 |
| *Haemoproteus* | Reduced.model | 0.479 | 0.322 | 0.628 |

**Table S6**. Standardized major axis (SMA) regression of body weight (log) on wing length (log) to estimate *b_SMA_* used for calculating body condition index. Reported are the scientific names of each host species, sample size (N), Slope of the fitted standardized major axis, lower and upper 95% confidence intervals (CI) and correlation coefficient (R^2^).

| **Species** | **N** | **Slope** | **Lower-95% CI** | **Upper-95% CI** | **R^2^** |
| --- | --- | --- | --- | --- | --- |
| *Alcippe poioicephala* | 53 | 2.385 | 1.748 | 3.255 | 0.061 |
| *Anthus nilghiriensis* | 12 | 2.134 | 1.198 | 3.801 | 0.308 |
| *Anthus rufulus* | 3 | 2.467 | 0.646 | 9.416 | 0.961 |
| *Culicicapa ceylonensis* | 25 | 2.100 | 1.388 | 3.176 | 0.080 |
| *Cyornis pallipes* | 17 | 1.528 | 0.880 | 2.654 | 0.023 |
| *Eumyias albicaudatus* | 40 | 1.848 | 1.307 | 2.615 | 0.000 |
| *Ficedula nigrorufa* | 89 | 1.872 | 1.498 | 2.341 | 0.022 |
| *Garrulax delesserti* | 27 | 1.856 | 1.327 | 2.595 | 0.263 |
| *Hypsipetes leucocephalus* | 53 | 1.449 | 1.089 | 1.927 | 0.181 |
| *Iole indica* | 21 | 2.009 | 1.448 | 2.785 | 0.697 |
| *Montecincla cachinnans* | 27 | 3.229 | 2.194 | 4.752 | 0.159 |
| *Montecincla fairbanki* | 84 | 1.879 | 1.510 | 2.339 | 0.039 |
| *Montecincla jerdoni* | 21 | 2.391 | 1.568 | 3.647 | 0.136 |
| *Montecincla meridionalis* | 33 | 2.041 | 1.401 | 2.975 | 0.021 |
| *Pellorneum ruficeps* | 23 | 2.199 | 1.462 | 3.308 | 0.198 |
| *Pomatorhinus horsfieldii* | 31 | 3.487 | 2.435 | 4.995 | 0.154 |
| *Pycnonotus jocosus* | 22 | 3.662 | 2.346 | 5.714 | 0.077 |
| *Rhopocichla atriceps* | 33 | 2.917 | 2.003 | 4.248 | 0.025 |
| *Saxicola caprata* | 15 | 3.467 | 2.203 | 5.457 | 0.137 |
| *Schoenicola platyurus* | 7 | 0.927 | 0.433 | 1.984 | 0.032 |
| *Sholicola albiventris* | 64 | 1.970 | 1.539 | 2.521 | 0.021 |
| *Sholicola ashambuensis* | 11 | 2.350 | 1.424 | 3.880 | 0.477 |
| *Sholicola major* | 79 | 1.643 | 1.298 | 2.078 | 0.042 |
| *Turdus merula* | 80 | -1.672 | -2.081 | -1.342 | 0.044 |
| *Zoothera citrina* | 6 | 6.963 | 2.934 | 16.527 | 0.106 |
| *Zoothera dauma* | 16 | -4.687 | -8.525 | -2.577 | 0.007 |
| *Zosterops palpebrosus* | 99 | 3.551 | 2.841 | 4.438 | 0.024 |

**FIGURES**

**Figure S1.** Fit between the observed and predicted probabilities of Haemosporidian infection for each bird species. Scatter plots are shown for: (a) *Plasmodium* and (b) *Haemoproteus.* Predicted infection probabilities were derived from the final reduced Bayesian phylogenetic mixed models (see Table S3). Symbols are sized relative to the sample size associated with the species, and error bars are bootstrapped 95% confidence intervals.


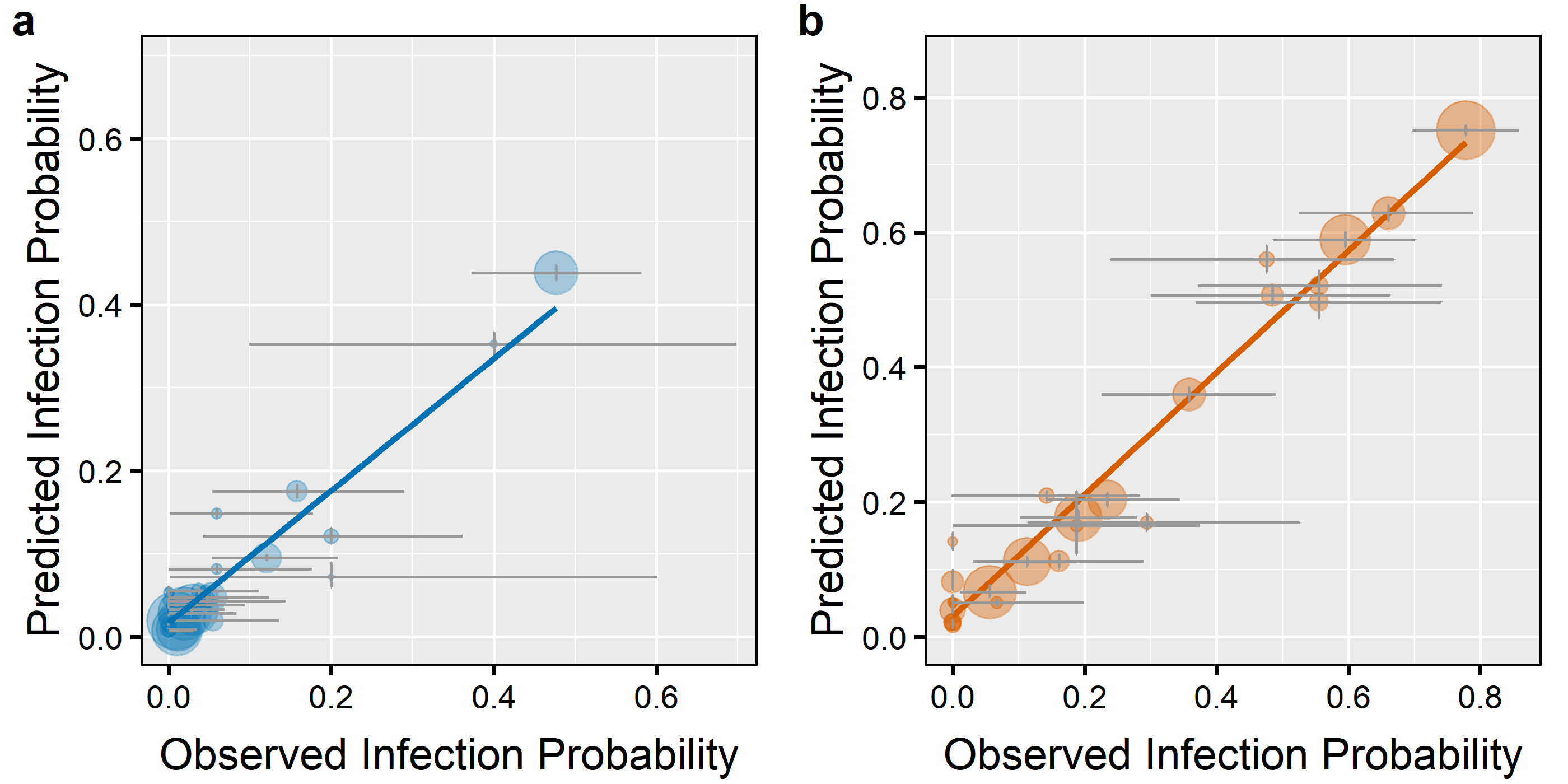


**REFERENCES**

1. Sasikumar C, Vishnudas CK, Raju S, Vinayan PA, Shebin VA. Malabar Ornithological Survey Report, 2010–2011. Trivandrum, Kerala Forest Department; 2011.

2. Robin V V, Sukumar R. Status and habitat preference of white-bellied shortwing Brachypteryx major in the Western Ghats (Kerala and Tamilnadu), India. Bird Conserv Int. 2002;12:335–52.

3. Robin VV, Sukumar R, Thiollay JM. Status and distribution of the White-bellied Shortwing Brachypteryx major in the Western Ghats of Karnataka and Goa, India. Bird Conserv Int. 2006;16:345–51.

4. Rajamani N, Nandini R, Mudappa D. Mystery or myth: a review of history and conservation status of the Malabar Civet Viverra civettina Blyth, 1862. Small Carniv. Conserv. 2010;43:47-59.

5. Lougheed SC, Arnold TW, Bailey RC. Measurement Error of External and Skeletal Variables in Birds and Its Effect on Principal Components. Auk. Oxford Academic; 1991;108:432–6.
